# Associations between lifestyle and well‐being in early and late pregnancy in women with overweight or obesity: Secondary analyses of the PEARS RCT

**DOI:** 10.1111/bjhp.12776

**Published:** 2025-01-16

**Authors:** Kaat Philippe, Alexander P. Douglass, Fionnuala M. McAuliffe, Catherine M. Phillips

**Affiliations:** ^1^ School of Public Health, Physiotherapy and Sports Science University College Dublin Dublin 4 Ireland; ^2^ UCD Perinatal Research Centre, National Maternity Hospital, School of Medicine University College Dublin Dublin 2 Ireland

**Keywords:** diet, healthy lifestyle score, overweight and obesity, physical activity, psychological well‐being, sleep, smoking

## Abstract

**Objectives:**

The associations between individual lifestyle behaviours and well‐being are still poorly understood, particularly in the antenatal period when women are exposed to physiological changes and increased psychological distress. A healthy lifestyle score (HLS) comprising protective lifestyle behaviours may be useful for studying links between overall lifestyle and psychosocial outcomes. This study aimed to examine bidirectional associations between a HLS and its components and psychological well‐being in pregnant women with overweight/obesity.

**Design:**

Secondary analyses of data from the PEARS trial.

**Methods:**

Healthy lifestyle scores (scored 0–5) based on maternal diet (AHEI‐P), physical activity (MET‐minutes), alcohol consumption, smoking, and sleep habits were created for 330 and 287 mothers with overweight/obesity in early (14–16 weeks gestation) and late pregnancy (28 weeks gestation), respectively. Psychological well‐being was measured with the WHO‐5 well‐being index. Cross‐lagged path models (crude/adjusted) tested the directionality of relationships between lifestyle (composite score/individual components) and well‐being cross‐sectionally and over time in pregnancy.

**Results:**

The mean early pregnancy BMI was 29.2 kg/m^2^. The mean well‐being score was 56.3% in early and 60.7% in late pregnancy. Significant autoregressive effects were observed for the HLS, all individual components, and well‐being from early to late pregnancy. Well‐being was positively correlated with the HLS, physical activity, and sleep variables within time points (in early and/or late pregnancy). Sleep and no smoking in early pregnancy predicted higher well‐being in late pregnancy.

**Conclusions:**

Overall healthy lifestyle, physical activity, and especially sleep duration and quality are associated with psychological well‐being in pregnancy, and should be promoted antenatally.

**Statement of Contribution**

**What Is Already Known on this Subject?**

- Composite healthy lifestyle scores, comprising low‐risk protective lifestyle behaviours may be useful for studying links between overall lifestyle and health outcomes.
- Maternal psychological distress is common in pregnancy.
- Women with overweight and obesity are at greater risk of psychological distress.

**What Does this Study Add?**

- To the best of our knowledge, this is the first study describing a prenatal composite healthy lifestyle score that includes sleep data.
- This study provides insight into the associations between lifestyle and well‐being throughout pregnancy among women with overweight and obesity, and the potential directionality of these associations.
- Overall healthy lifestyle (reflected with the healthy lifestyle score), physical activity, and especially sleep duration and quality are associated with psychological well‐being in pregnancy.

## INTRODUCTION

A growing body of evidence demonstrates the importance of prenatal lifestyle for maternal and offspring health. To illustrate, smoking during pregnancy has been consistently associated with adverse birth outcomes including stillbirth and low birth weight (Avşar et al., 2021). Poor dietary quality has been associated with smaller birth size and a higher risk of being born small for gestational age (Chen et al., 2021). Engaging in moderate‐intensity physical activity during pregnancy can reduce the risk of excessive maternal weight gain and gestational diabetes mellitus (Dipietro et al., 2019). Disturbances in sleep, a lifestyle factor that is often overlooked, have been associated with pregnancy complications including pre‐eclampsia, gestational hypertension and gestational diabetes mellitus, caesarean section, preterm birth, large for gestational age, and stillbirth (Lu et al., 2021). Composite measures of a healthy lifestyle, such as healthy lifestyle scores (HLS), based on low‐risk behaviours (e.g. no smoking, good diet quality, and sufficient levels of physical activity) can be useful for studying links between overall lifestyle and health outcomes. They potentially have more predictive power than single behaviours alone (Sotos‐Prieto et al., 2015), due to synergistic effects, and have previously been linked with more favourable cardiometabolic health outcomes and life expectancy (Díaz‐Gutiérrez et al., 2018; Li et al., 2018; Millar et al., 2022). In pregnant populations, emerging research indicates prenatal HLS associations with offspring birth outcomes and childhood obesity (Navarro et al., 2020). HLSs are believed to be a useful screening tool in preventive care, which can stimulate more holistic behavioural approaches (Sotos‐Prieto et al., 2015).

Lifestyle behaviours have also been studied in relation to psychological well‐being and psychopathology, but the evidence is still limited, especially in pregnancy. Poor sleep quality and sub‐optimal sleep duration in pregnancy have been associated with higher scores for depression, anxiety, and stress (review by Pauley et al., 2020). Physical activity during pregnancy has been positively associated with psychological well‐being (Da Costa et al., 2003; Horan et al., 2014), while the associations between prenatal diet quality and well‐being and psychopathology are currently inconsistent (Horan et al., 2014; Sparling et al., 2017). Thus far, no research has examined composite HLSs and well‐being during pregnancy.

Given that maternal psychological distress (e.g. depression, anxiety, stress) is common in pregnancy (Priest et al., 2008; Woods et al., 2010), it is worthwhile to investigate the associations between lifestyle during pregnancy and psychological well‐being, as they could impact both maternal and child health pre‐ and postnatally. Questions also remain about the directionality between lifestyle and well‐being, both in non‐pregnant and pregnant populations. Most studies on this topic are cross‐sectional, with data collected at one‐time point. Some studies, with longitudinal data, have provided evidence for considering an effect of lifestyle on well‐being or vice versa. For example, a follow‐up study of a middle‐aged and elderly population in Lithuania demonstrated a positive association between healthy lifestyle behaviours (not smoking, fruit and vegetable consumption, physical activity) and higher psychological well‐being scores 10 years later (Sapranaviciute‐Zabazlajeva et al., 2017). The release of endorphins following physical activity and modulated brain chemistry in relation to microbiota have been suggested as potential contributors to increased well‐being (Dinan & Cryan, 2013; Fox, 1999). In the other direction, anxiety and depression symptoms among women have been associated with reduced odds of having a healthy lifestyle 20 years later (Trudel‐Fitzgerald et al., 2016). Roche et al. (2023) showed that well‐being during pregnancy significantly influenced the positive stage of behaviour change and could act as a lever to engage in healthy lifestyle behaviours during pregnancy. Both directionalities can therefore be considered plausible.

Overweight and obesity are well‐recognised risk factors for depression, anxiety, and other mental health conditions (Azarbad & Gonder‐Frederick, 2010). Rates of overweight and obesity are currently also rising among women of reproductive age (Poston et al., 2016), making it an increasingly common feature during pregnancy, and thus a relevant concern in the prenatal care setting. Moreover, as pregnancy can be psychologically challenging, women with overweight/obesity may be at greater risk of distress and lower well‐being.

Therefore, the aim of this exploratory study is to investigate the potential bidirectional associations between maternal lifestyle factors (diet quality, physical activity, smoking, alcohol consumption, and sleep) individually and as a composite HLS, and psychological well‐being in early and late pregnancy in women with overweight/obesity.

## MATERIALS AND METHODS

### Study design and participants

This study involved secondary analyses of data collected for the Pregnancy Exercise And nutrition Research Study (PEARS). This was a single‐centre randomised controlled trial of a diet and exercise lifestyle intervention with smartphone application support to prevent gestational diabetes mellitus among women with overweight/obesity (trial registration number: ISRCTN29316280). The trial received ethical approval from The National Maternity Hospital Ethics Committee in October 2012.

Women were eligible for enrolment if they were aged 18–45 years, at 10–18 weeks' gestation with a singleton pregnancy, had a BMI ≥25 kg/m^2^ and ≤39.9 kg/m^2^ at recruitment, and were in possession of a smartphone. Reasons for exclusion included previous gestational diabetes mellitus and any medical condition requiring treatment. Data collection took place between 2013 and 2016. Participants were randomised into the diet and physical activity intervention group or control group with standard antenatal care. The intervention took place from the first antenatal appointment until 34 weeks of gestation. Participants in the intervention groups received in‐person diet and physical activity education sessions in small groups, fortnightly personalised emails, and access to the study‐specific smartphone app with information and low glycaemic index recipes. Details of the PEARS trial and outcome results have been published elsewhere (Kennelly et al., 2016, 2018). In brief, the intervention did not decrease the incidence of gestational diabetes mellitus (Kennelly et al., 2018), but did improve dietary intakes, physical activity, and motivation to engage in exercise among participants (Ainscough et al., 2020).

For the current secondary analyses, only those participants with a complete HLS and a well‐being score in early or late pregnancy available were included (*n* = 387). All these women were considered completers from the intervention or control group, even if they had not completed all surveys at each timepoint. Women who did not attend the initial visit (*n* = 18) or discontinued their participation (*n* = 49) were not considered for the current analysis because of the medically related causes (e.g., miscarriage, congenital anomalies) or the uncertainty about the causes–which may impact our variables of interest (lifestyle and well‐being). A detailed flow‐chart of the participants can be found in the Supporting Information (Table S1), as well as a comparison of included and excluded participants (Table S2).

### Measures

HLSs in most previous research have comprised of the following behaviours/factors: diet, physical activity, smoking, alcohol consumption, and body mass index (BMI) (e.g., Li et al., 2018; Navarro et al., 2020). This study will build on this previous research with some adaptations in function of the current research aim and population. First, as the PEARS study population are pregnant women with overweight/obesity (determined at recruitment in early pregnancy), BMI was not included in the HLS in this study but treated as a covariate in the analyses. Second, sleep information was included as part of the HLS, because of its known associations with prenatal psychological distress. In a non‐pregnant population, Guasch‐Ferré et al. (2022) have already demonstrated the usefulness of the inclusion of a sleep component as part of a HLS in relation to cardiovascular disease. The following lifestyle variables (individually and as part of a composite HLS) were thus of key interest in this study: diet quality, physical activity, smoking status, alcohol consumption, and sleep duration and quality, alongside psychological well‐being.

Lifestyle and well‐being data were collected twice: at the first antenatal appointment (14–16 weeks gestation) and in late pregnancy (28 weeks gestation). Participants were instructed to complete lifestyle questionnaires with questions about physical activity, smoking status, alcohol intake, sleep duration, and well‐being. Using a 3 days food diary, they were also asked to record all food and beverages consumed during 2 weekdays and 1 weekend day to assess normal eating habits.

#### Diet quality

Overall diet quality during pregnancy was assessed using the Alternate Healthy Eating Index modified for Pregnancy (AHEI‐P) (Rodríguez‐Bernal et al., 2010). Participants recorded the types and amount of food consumed in their food diary in household measures (e.g. teaspoons, tablespoons) or using the weight listed on food and beverage packaging. The AHEI‐P is based on a 100‐point scale with 0–10 points awarded for optimal intake of 10 types of foods and nutrients (components). Participants received higher scores for higher intakes of healthier components (vegetables, fruit, cereal fibre, nuts/soy/vegetable protein, folate, calcium, and iron). For every 10% decrease in intake, 1 point is subtracted. Participants also received higher scores for higher ratios of white–red meat and polyunsaturated–saturated fatty acids with scores increasing proportionate to these ratios. Conversely, they received lower scores for higher percentage intake of trans‐fats relative to total energy intake (Rodríguez‐Bernal et al., 2010).

#### Physical activity

Physical activity was reported with an adapted item from the Irish 2002 Survey of Lifestyle, Attitudes and Nutrition (SLÁN, Kelleher et al., 2003) which has been validated for use in pregnancy (Walsh et al., 2012). Participants were asked to report the frequency of 30 min intervals of mild (‘minimal effort’, e.g. easy walking, light gardening), moderate (‘not exhausting’, e.g. easy cycling/swimming) and vigorous (‘hearts beat rapidly’, e.g. running, vigorous swimming) leisure time activity over a 7 days period. Using their answers, metabolic equivalents of task (MET) minutes per week were calculated (Ainsworth & Swartz, 2000).

#### Smoking

Smoking behaviour was assessed with items from the 2002 SLÁN (Kelleher et al., 2003). Participants declared if they were currently smoking (regularly or occasionally) or not. They also provided information about the number of cigarettes smoked and past smoking habits but this last information was not used in the current study.

#### Alcohol consumption

Alcohol consumption was assessed using one item from the 2002 SLÁN (Kelleher et al., 2003). Participants declared when they had last consumed an alcoholic drink: in the last week, 1 week–1 month ago, 1 month–3 months ago, 3 months–12 months ago, more than 12 months ago, or never had alcohol beyond taste and sips. For the current analyses investigating lifestyle in early and late pregnancy and their associations, a binary variable was created to distinguish participants who had not been recently drinking (last alcoholic drink >3 months ago) from participants who had been drinking in the last 3 months.

#### Sleep

Sleep duration and sleep quality were assessed using two items from the Pittsburgh Sleep Quality Index (Buysse et al., 1989) which has been validated in a pregnant population (Facco et al., 2010). Participants self‐reported their estimated sleep duration in hours (‘During the past month, how many hours of actual sleep did you get at night?’) and were asked to rate their overall sleep quality during the past month on a four‐point scale (very good–fairly good–fairly bad–very bad). A higher score indicates higher self‐perceived sleep quality.

#### Healthy lifestyle score

A HLS was calculated for each participant based on their adherence to the recommendations regarding five lifestyle behaviours: diet quality, physical activity, smoking, alcohol consumption, and sleep duration. In line with previous research (Guasch‐Ferré et al., 2022; Li et al., 2018; Navarro et al., 2020), participants received a score of 1 when they adhered to current recommendations for a certain behaviour, or a score of 0 if they did not. The recommendations state that adults, including healthy pregnant adults, should engage in a minimum of 150 minutes of moderate‐intensity aerobic activity per week (American College of Obstetricians and Gynaecologists, 2020), which equals 500 MET‐minutes or more per week (2018 Physical Activity Guidelines Advisory Committee, 2018), sleep 7–9 h per night (Hirshkowitz et al., 2015), and not smoke or consume alcohol during pregnancy (Koletzko et al., 2014). Having an AHEI‐P score in the top 40% of a study population indicates a recommended diet quality (Navarro et al., 2020). Scores for each individual behaviour were summed, generating a HLS (range 0–5), with higher scores indicating adherence to a higher number of healthy lifestyle behaviours and thus a healthier overall lifestyle.

#### Psychological general well‐being

Participants' ‘psychological general well‐being’, often referred to as ‘subjective well‐being’, was assessed in this study, which is a more generic indication of mental health, exploring positive moods, vitality, and general interests, without any diagnostic specificity (Bech, 2004; Bech et al., 1996). It was assessed using the World Health Organization's Well‐being Index (WHO‐5) (Bech, 2004), which has been widely validated and has demonstrated adequate validity both as a screening tool for depression and as an outcome measure in clinical trials (Topp et al., 2015). Participants provided information about their feelings or activities over the past 2 weeks through five items, for example, ‘I have felt cheerful and in good spirits’, ‘My daily life has been filled with things that interested me’. Scores for each statement ranged from 0 (‘at no time’) to 5 (‘all the time’). Total well‐being scores range from 0 to 25, with higher scores indicating a higher well‐being. The total score was transformed to a scale of 0–100 to facilitate interpretability. The internal consistency of this scale was good with a Cronbach's alpha of 0.77 in early pregnancy and 0.81 in late pregnancy.

### Covariates

Based on the literature, the following covariates were considered in the analyses: maternal BMI at recruitment, parity, maternal age at recruitment, neighbourhood deprivation as an indicator of socio‐economic status, and group (intervention vs. control). Demographic information was self‐reported at the initial visit and all participants were measured and weighed. Neighbourhood deprivation data were obtained through the Pobal Haase‐Pratschke Deprivation Index (HP index) address‐mapping tool (Haase & Pratschke, 2017), which integrates information about 10 measures of an area's levels of disadvantage, including educational attainment, employment status and the numbers living in individual households (Haase & Pratschke, 2017).

### Statistical analyses

All statistical analyses were conducted with Stata SE 18. Descriptive characteristics and scores were examined according to the number of health behaviours (healthy lifestyle score) during early and late pregnancy. Participants were grouped into three HLS groups (score 0–2, score 3, and score 4–5) to ensure sufficient numbers of participants in each group, as there were only few participants with an HLS of 0, 1, or 5 in early and late pregnancy (score 0: 0.3%/0.35% in early/late pregnancy; score 1: 6.06%/4.18%; score 5: 10.3%/8.36%, respectively–see Supporting Information Table S3). One‐way ANOVA or Chi‐square was computed to determine overall differences in characteristics between these groups (presented with *p*‐values).

Correlations as well as changes in main study variables from early to late pregnancy were explored. The results of these analyses are presented in the Supporting Information (Tables S4–S6).

**FIGURE 1:**
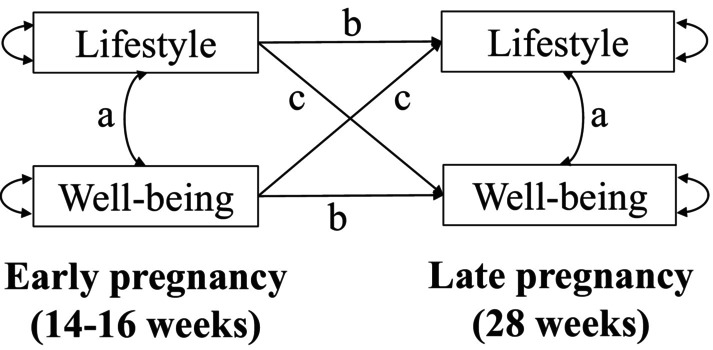
Conceptual model of the cross‐lagged panel models. The letter (a) signifies the correlations within time points, (b) the autoregressive effects over time, and (c) the crossed‐lagged paths.

Cross‐lagged panel models were used to describe the reciprocal relationships between lifestyle and well‐being from early to late pregnancy. This is a type of structural equation modelling that has been proven to be particularly useful for examining the stability and relationships between variables over time (Kearney, 2017). The models allow for the estimation of cross‐lagged effects (i.e., the effect of one variable at timepoint 1 on another variable at timepoint 2) while also controlling for correlations within timepoints (i.e. the association between different variables at the same timepoint) and autoregressive effects over time (i.e. the effect of a variable on itself from timepoint 1 to timepoint 2). Separate models with maximum likelihood of missing data were run for each lifestyle behaviour and the HLS so all available data could be used in the models. Figure 1 depicts the conceptual model of the cross‐lagged panel models analysed. In a first stage, simple models were analysed without adjusting for covariates (examining associations between one lifestyle behaviour and well‐being in early and late pregnancy). In a second stage, the models were adjusted for maternal BMI at recruitment, parity, maternal age at recruitment, neighbourhood deprivation (HP index), and group (intervention vs. control), by adding a direct effect of these covariates on each of the lifestyle and well‐being variables in the models in early and late pregnancy. For the HLS, diet, physical activity, sleep duration and sleep quality, the continuous variables were used in the models. For smoking and alcohol consumption, the categorical dichotomous variables were used. Comparative analyses (Chi‐square) were also run to test for significant differences between standardised path coefficients of the cross‐lagged paths.

Furthermore, sensitivity analyses were carried out after the exclusion of participants with gestational diabetes mellitus or pre‐eclampsia (*n* = 64), as these conditions can potentially influence pregnant women's lifestyle and well‐being. Both unadjusted and adjusted sensitivity analyses were conducted, using the same statistical approach as in the main analyses described above.

Additional sensitivity analyses were conducted on a subset of women with BMI data available in both early and late pregnancy (*n* = 152), which allowed adjustment for change in BMI from recruitment to 28 weeks of gestation by adding a direct effect of change in BMI on each of the lifestyle and well‐being variables in the models in late pregnancy (additional to the other covariates). The results of these models were compared with models that did not additionally adjust for change in BMI.

To correct for the multiple testing and reduce the risk of Type I errors, the Benjamini and Hochberg correction for multiple testing (Benjamini & Hochberg, 1995) was applied for the main and sensitivity analyses with a false discovery rate (FDR) of 0.05.

## RESULTS

### Characteristics of study participants

Table 1 describes the characteristics of the participants at early (*n* = 330) and late pregnancy (*n* = 287), overall and according to their HLS. In early pregnancy, 26% of women had a HLS of 0–2 (‘low’), meaning they adhered to maximum 2 out of 5 healthy lifestyle behaviours, 41% adhered to 3 healthy lifestyle behaviours in early pregnancy (‘moderate’), and 33% had a HLS of 4 or 5 (‘high’). In late pregnancy, 30% of women had a low HLS, 33% a moderate HLS, and 36% a high HLS. Note that in this last group, the percentage of women in the diet and exercise intervention group was significantly higher. Significant differences were observed in early and/or late pregnancy for parity, level of education, and ethnicity in relation to the number of healthy behaviours adhered to. No differences in age, BMI, or social (dis)advantage were observed in early and late pregnancy. Individual lifestyle factors were more favourable among women with higher HLSs, except for early pregnancy alcohol consumption among women with a moderate HLS. Well‐being scores were also higher on average in women with a higher HLS.

**TABLE 1:** Maternal characteristics and mean lifestyle and well‐being scores in early and late pregnancy: Overall and according to healthy lifestyle score (HLS) group.

	Early pregnancy (14–16 weeks gestation)				*p*_trend_ ^e^	Late pregnancy (28 weeks gestation)				*p*_trend_ ^e^
	All	HLS 0–2	HLS 3	HLS 4–5		All	HLS 0–2	HLS 3	HLS 4–5	
	(*n* = 330)	(*n* = 86)	(*n* = 136)	(*n* = 108)		(*n* = 287)	(*n* = 87)	(*n* = 96)	(*n* = 104)	
Maternal characteristics										
Age at recruitment (M ± SD)	32.3 ± 4.2	32.7 ± 4.8	32.9 ± 4.0	32.3 ± 4.0	.557	32.6 ± 4.2	32.8 ± 3.9	32.7 ± 4.3	32.2 ± 4.3	.559
Baseline BMI (kg/m^2^) (M ± SD)	29.4 ± 3.3	29.8 ± 3.8	29.4 ± 3.3	29.0 ± 2.9	.242	29.2 ± 3.3	29.7 ± 3.6	28.9 ± 3.0	29.0 ± 3.1	.243
Having previous child (ren) (%)	43.0%	43.0%	42.7%	32.4%	.**003**	43.9%	41.4%	36.5%	38.5%	.**004**
Tertiary education or higher (%)	67.0%	57.0%	64.0%	78.7%	.**024**	69.0%	66.7%	68.8%	71.2%	.627
Living in advantaged area^a^ (%)	42.7%	31.4%	45.6%	48.2%	.153	41.1%	36.8%	42.7%	43.3%	.908
White ethnicity (%)	91.5%	94.2%	88.9%	92.3%	.892	91.3%	87.4%	88.4%	97.1%	.**047**
In intervention group (%)	50.3%	55.8%	47.8%	49.1%	.484	47.7%	42.5%	39.6%	59.6%	.**009**
Lifestyle and well‐being scores										
Not currently smoking (%)	95.5%	87.2%	97.1%	100.0%	.**000**	95.5%	87.4%	97.9%	100.0%	.**000**
No alcohol consumption in past 3 months (%)	61.2%	67.4%	55.9%	90.7%	.**000**	77.0%	58.6%	82.3%	87.5%	.**000**
Diet: AHEI‐P^b^ (M ± SD)	54.5 ± 10.5	48.9 ± 7.7	53.4 ± 9.8	60.3 ± 10.6	.**000**	55.0 ± 10.7	49.1 ± 8.8	54.9 ± 11.0	60.0 ± 9.2	.**000**
Physical activity in MET‐minutes (M ± SD)	498.5 ± 441.2	252.2 ± 284.2	455.9 ± 442.0	748.1 ± 417.0	.**000**	524.8 ± 389.7	296.2 ± 285.3	486.4 ± 331.6	751.6 ± 393.6	.**000**
Sleep duration in minutes (M ± SD)	437.8 ± 73.5	404.8 ± 88.1	441.8 ± 70.4	459.0 ± 53.1	.**000**	393.1 ± 74.6	350.3 ± 66.9	389.8 ± 68.4	432.0 ± 65.8	.**000**
Sleep quality^c^ (M ± *SD*)	2.9 ± 0.7	2.6 ± 0.8	2.9 ± 0.7	3.0 ± 0.6	.**000**	2.7 ± 0.7	2.4 ± 0.7	2.6 ± 0.7	2.9 ± 0.7	.**000**
Well‐being score^d^ (M ± *SD*)	56.3 ± 15.2	51.5 ± 15.7	55.4 ± 15.1	61.3 ± 13.4	.**000**	60.7 ± 14.8	55.8 ± 15.5	61.0 ± 13.4	64.4 ± 14.4	.**000**

*Note*: HLS = healthy lifestyle score, ranging from 0 to 5 with higher scores indicating a higher adherence to healthy lifestyle recommendations.

^a^ Living in an advantaged area is defined as having a HP index above 10.

^b^ AHEI‐P = alternate healthy eating index–adapted for pregnancy with scores ranging from 0 to 100 with higher scores indicating a higher diet quality; MET = metabolic equivalents of task.

^c^ Sleep quality is a categorical variable (1–2–3‐4) with higher scores indicating a better self‐perceived sleep quality.

^d^ Well‐being score ranging from 0 to 100 with higher scores indicating a higher well‐being.

^e^ Significant (*p* < .05) differences in characteristics according to HLS group are in bold.

### Cross‐lagged path models

**FIGURE 2:**
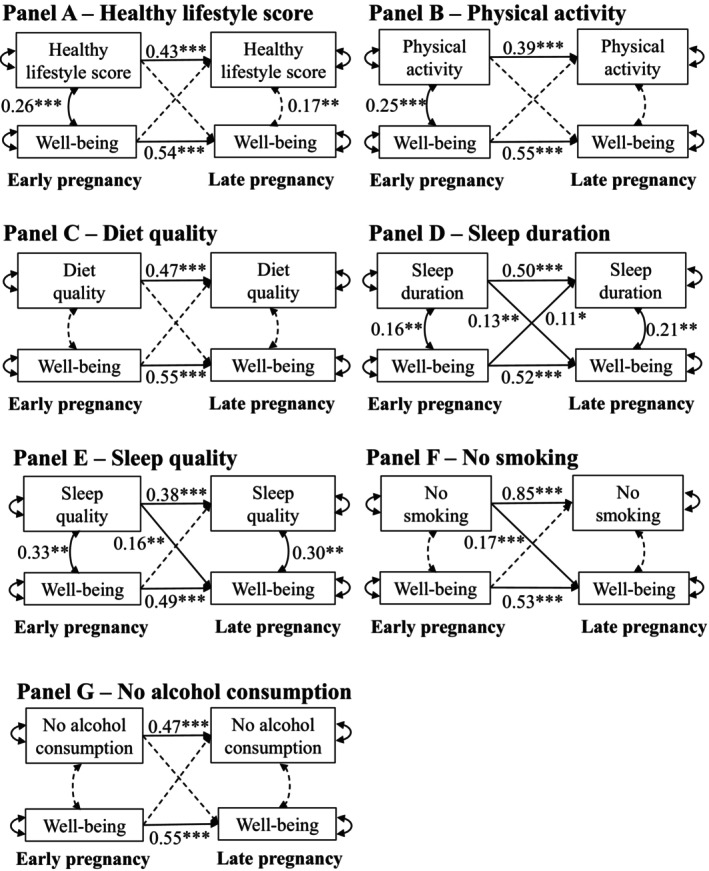
Results of the cross‐lagged path models investigating the associations between well‐being in early and late pregnancy and health behaviours. Panel (a) shows the model including the composite healthy lifestyle score, Panel (b) physical activity, Panel (c) diet quality, Panel (d) sleep duration, Panel (e) sleep quality, Panel (f) not smoking, and Panel (g) no alcohol consumption. The healthy lifestyle score, physical activity, diet quality, sleep duration, sleep quality, and well‐being were entered in the models as continuous variables, no smoking and no alcohol as dichotomous categorical variables. All models were adjusted for maternal BMI and age at recruitment, parity, neighbourhood deprivation, and intervention group (covariates not shown in the panels to enhance readability; results are presented in the Supporting Information (Table S10)). Significant path estimates (*p* < .05) are depicted with a full line, non‐significant paths (*p* ≥ .05) with dotted lines. ***: *p* < .001, **: *p* < .01, *: *p* < .05. All coefficients are standardised. *R* ^2^‐values, indicating total variance explained on the outcome, ranged between .25 and 0.49 in the models (see Table S8).

Results from the unadjusted and adjusted cross‐lagged path models presented in Table 2 were very similar in terms of significance (*p* < 0.05) and directionality. Figure 2 illustrates the results of the adjusted cross‐lagged path models discussed below. Associations between the covariates and the lifestyle and well‐being variables are only shown in the Supporting Information(Table S10).

**TABLE 2:** Results of the cross‐lagged path models based on all participants (*n* = 387).

	Unadjusted path models			Adjusted^a^ path models		
	Estimate^b^ (95% CI)	*SE*	*p*‐value	Estimate^b^ (95% CI)	*SE*	*p*‐value
Correlations within time points						
Early healthy lifestyle score–Early well‐being	0.27 (0.17, 0.37)	0.05	.**000**	0.26 (0.16, 0.36)	0.05	.**000**
Early physical activity–Early well‐being	0.25 (0.16, 0.35)	0.05	.**000**	0.25 (0.16, 0.35)	0.05	.**000**
Early diet–Early well‐being	0.10 (−0.00, 0.20)	0.05	.062	0.09 (−0.01, 0.20)	0.05	.072
Early sleep duration–Early well‐being	0.16 (0.05, 0.26)	0.05	.**003**	0.16 (0.05, 0.26)	0.05	.**003**
Early sleep quality–Early well‐being	0.33 (0.24, 0.43)	0.05	.**000**	0.33 (0.24, 0.42)	0.05	.**000**
Early no smoking–Early well‐being	0.10 (−0.00, 0.20)	0.05	.062	0.10 (−0.00, 0.20)	0.05	.058
Early no alcohol–Early well‐being	0.02 (−0.09, 0.12)	0.05	.729	0.02 (−0.08, 0.12)	0.05	.711
Late healthy lifestyle score–Late well‐being	0.21 (0.10, 0.32)	0.06	.**000**	0.17 (0.06, 0.29)	0.06	.**003**
Late physical activity–Late well‐being	0.13 (0.01, 0.24)	0.06	**.033** ^d^	0.09 (−0.02, 0.21)	0.06	.121
Late diet–Late well‐being	0.10 (0.02, 0.21)	0.06	.111	0.08 (−0.04, 0.20)	0.06	.191
Late sleep duration–Late well‐being	0.23 (0.12, 0.34)	0.06	.**000**	0.21 (0.10, 0.32)	0.06	.**000**
Late sleep quality–Late well‐being	0.33 (0.23, 0.43)	0.05	.**000**	0.30 (0.20, 0.41)	0.05	.**000**
Late no smoking–Late well‐being	0.09 (−0.03, 0.20)	0.06	.129	0.07 (−0.05, 0.18)	0.06	.245
Late no alcohol–Late well‐being	−0.04 (−0.16, 0.07)	0.06	.465	−0.09 (−0.20, 0.03)	0.06	.129
Autoregressive effects over time						
Early healthy lifestyle score–Late healthy lifestyle score	0.42 (0.31, 0.53)	0.06	.**000**	0.43 (0.32, 0.54)	0.06	.**000**
Early physical activity–Late physical activity	0.43 (0.33, 0.53)	0.05	.**000**	0.39 (0.30, 0.49)	0.05	.**000**
Early diet–Late diet	0.48 (0.38, 0.57)	0.05	.**000**	0.47 (0.38, 0.56)	0.05	.**000**
Early sleep duration–Late sleep duration	0.51 (0.42, 0.60)	0.05	.**000**	0.50 (0.41, 0.59)	0.05	.**000**
Early sleep quality–Late sleep quality	0.39 (0.29, 0.50)	0.05	.**000**	0.38 (0.28, 0.49)	0.05	.**000**
Early no smoking–Late no smoking	0.85 (0.82, 0.88)	0.02	.**000**	0.85 (0.81, 0.88)	0.02	.**000**
Early no alcohol–Late no alcohol	0.47 (0.38, 0.57)	0.05	.**000**	0.47 (0.38, 0.56)	0.05	.**000**
Early well‐being–Late well‐being^c^	Ranging between 0.51–0.58	0.04–0.05	**All .000**	Ranging between 0.49–0.55	0.04–0.05	**All .000**
Cross‐lagged paths						
Early healthy lifestyle score–Late well‐being	0.06 (−0.04, 0.17)	0.05	.235	0.04 (−0.07, 0.15)	0.05	.453
Early physical activity–Late well‐being	0.01 (−0.09, 0.12)	0.05	.775	−0.02 (−0.12, 0.08)	0.05	.693
Early diet–Late well‐being	0.01 (−0.10, 0.11)	0.05	.909	0.01 (−0.09, 0.11)	0.05	.826
Early sleep duration–Late well‐being	0.14 (0.04, 0.24)	0.05	.**005**	0.14 (0.04, 0.23)	0.05	.**004**
Early sleep quality–Late well‐being	0.19 (0.09, 0.29)	0.05	.**000**	0.16 (0.07, 0.26)	0.05	.**001**
Early no smoking–Late well‐being	0.19 (0.10, 0.28)	0.05	.**000**	0.17 (0.08, 0.26)	0.05	.**000**
Early no alcohol–Late well‐being	−0.05 (−0.15, 0.04)	0.05	.272	−0.06 (−0.15, 0.04)	0.05	.251
Early well‐being–Late healthy lifestyle score	0.04 (−0.04, 0.16)	0.06	.542	0.01 (−0.11, 0.13)	0.06	.879
Early well‐being–Late physical activity	0.05 (−0.06, 0.16)	0.06	.367	0.04 (−0.06, 0.15)	0.05	.414
Early well‐being–Late diet	0.00 (−0.10, 0.10)	0.05	.998	−0.02 (−0.12, 0.08)	0.05	.656
Early well‐being–Late sleep duration	0.13 (0.02, 0.23)	0.05	.**018**	0.11 (0.01, 0.21)	0.05	**.037** ^d^
Early well‐being–Late sleep quality	0.12 (0.01, 0.23)	0.06	**.035** ^d^	0.10 (−0.01, 0.22)	0.06	.065
Early well‐being–Late no smoking	−0.03 (−0.09, 0.04)	0.03	.419	−0.03 (−0.09, 0.03)	0.03	.342
Early well‐being–Late no alcohol	−0.03 (−0.14, 0.07)	0.05	.549	−0.04 (−0.14, 0.06)	0.05	.475

*Note*: Early = in early pregnancy (14–16 weeks gestation), Late = in late pregnancy (28 weeks gestation). Significant p‐values (p < .05) are in bold.

^a^ Only the results of the associations between lifestyle and well‐being are presented here. The healthy lifestyle score, physical activity, diet quality, sleep duration, sleep quality, and well‐being were entered in the models as continuous variables, no smoking and no alcohol as dichotomous categorical variables. The results of the associations with the covariates (maternal BMI and age at recruitment, parity, neighbourhood deprivation, and intervention group) in the adjusted models are not presented to enhance readability. These results are presented in the Supporting Information (Table S10).

^b^ All coefficients are standardised.

^c^ Range of values provided for the autoregressive effect between well‐being from early to late pregnancy because this effect was estimated in each path model–see bottom part of each Panel in Figure 2.

^d^ These *p*‐values became non‐significant after applying Benjamini–Hochberg corrections (corrected *p* = .063, .065, and .067, respectively).

#### Autoregressive effects over time

In all adjusted models, the autoregressive effects over time (same variable: well‐being/HLS/lifestyle behaviours, from early to late pregnancy) were significant (all *p* < .001). The lowest autoregressive effect was found for sleep quality (Coeff. = 0.38; Figure 2–Panel e) while the highest was found for no smoking (Coeff. = 0.85; Panel f).

#### Correlations within time points

Significant correlations within time points (between HLS/lifestyle behaviours and well‐being) were observed in the models with the HLS (Figure 2–Panel a), physical activity (Panel b), sleep duration (Panel d), and sleep quality (Panel e). For physical activity, the correlation with well‐being was significant in early pregnancy (*p* = .000), for HLS, sleep quality, and duration, they were significant in both early and late pregnancy.

#### Cross‐lagged paths

Significant cross‐lagged paths (between HLS/lifestyle behaviours and well‐being, from early to late pregnancy) were observed in the adjusted models with sleep duration (Figure 2–Panel d), sleep quality (Panel e), and no smoking (Panel f). Sleep quality and no smoking in early pregnancy both positively predicted well‐being in late pregnancy (*p* = .001 and *p* = .000 respectively). Sleep duration in early pregnancy significantly positively predicted well‐being in late pregnancy and vice versa (*p* = .004 and *p* = .037 respectively), although this last association did not withstand correction for multiple testing (corrected *p* = .067). All other associations in the adjusted models withstood correction for multiple testing.

Testing for significant differences between the standardised coefficients of the cross‐lagged paths only demonstrated significant differences in the paths of the no smoking model (unadj. model: Chi‐square = 13.78, *p* = .003, adj. model: Chi‐square = 12.61, *p* = .0004). See Table S9 for all results.

### Sensitivity analyses

The unadjusted and adjusted cross‐lagged path models were rerun excluding women with a diagnosis of gestational diabetes mellitus or pre‐eclampsia (*n* = 64). The results are presented in Table 3. Statistical significance and direction of results remained for the adjusted models including HLS, physical activity, no smoking, and no alcohol consumption. Differences were observed in the models including diet quality, sleep quality, and duration. The correlation between diet quality and well‐being in early pregnancy (Coeff. = 0.16, *p* = .003) and the cross‐lagged path between early pregnancy well‐being and late pregnancy sleep quality became significant (Coeff. = 0.14, *p* = .014), whereas the cross‐lagged paths between well‐being in early pregnancy and sleep duration in late pregnancy became non‐significant (Coeff. = 0.11, *p* = .054). All associations in the adjusted models withstood correction for multiple testing.

**TABLE 3:** Sensitivity analyses of cross‐lagged path models based on uncomplicated pregnancies (*n* = 323: Women without gestational diabetes mellitus or pre‐eclampsia).

	Unadjusted path models			Adjusted^a^ path models		
	Estimate^b^ (95% CI)	*SE*	*p*‐value	Estimate^b^ (95% CI)	*SE*	*p*‐value
Correlations within time points						
Early healthy lifestyle score–Early well‐being	0.28 (0.17, 0.39)	0.06	.**000**	0.28 (0.17, 0.38)	0.06	.**000**
Early physical activity–Early well‐being	0.26 (0.15, 0.36)	0.05	.**000**	0.26 (0.15, 0.36)	0.05	.**000**
Early diet–Early well‐being	0.17 (0.06, 0.28)	0.06	.**003**	0.16 (0.05, 0.27)	0.06	.**003**
Early sleep duration–Early well‐being	0.14 (0.02, 0.25)	0.06	.**018**	0.13 (0.02, 0.25)	0.06	.**019**
Early sleep quality–Early well‐being	0.32 (0.22, 0.42)	0.05	.**000**	0.32 (0.22, 0.42)	0.05	.**000**
Early no smoking–Early well‐being	0.08 (−0.03, 0.20)	0.06	.155	0.08 (−0.03, 0.20)	0.06	.153
Early no alcohol–Early well‐being	0.02 (−0.10, 0.13)	0.06	.787	0.02 (−0.10, 0.13)	0.06	.763
Late healthy lifestyle score–Late well‐being	0.18 (0.05, 0.30)	0.06	.**005**	0.15 (0.02, 0.28)	0.06	.**019**
Late physical activity–Late well‐being	0.10 (−0.03, 0.23)	0.07	.117	0.08 (−0.05, 0.21)	0.07	.223
Late diet–Late well‐being	0.08 (−0.05, 0.21)	0.06	.224	0.06 (−0.07, 0.19)	0.07	.348
Late sleep duration–Late well‐being	0.24 (0.12, 0.36)	0.06	.**000**	0.21 (0.09, 0.33)	0.06	.**000**
Late sleep quality–Late well‐being	0.34 (0.23, 0.45)	0.06	.**000**	0.31 (0.20, 0.42)	0.06	.**000**
Late no smoking–Late well‐being	0.09 (−0.03, 0.21)	0.06	.157	0.07 (−0.06, 0.19)	0.06	.286
Late no alcohol–Late well‐being	−0.04 (−0.16, 0.08)	0.06	.531	−0.07 (−0.20, 0.05)	0.06	.240
Autoregressive effects over time						
Early healthy lifestyle score–Late healthy lifestyle score	0.40 (0.28, 0.53)	0.06	.**000**	0.42 (0.31, 0.55)	0.06	.**000**
Early physical activity–Late physical activity	0.45 (0.34, 0.55)	0.05	.**000**	0.41 (0.31, 0.51)	0.05	.**000**
Early diet–Late diet	0.48 (0.39, 0.58)	0.05	.**000**	0.48 (0.38, 0.57)	0.05	.**000**
Early sleep duration–Late sleep duration	0.52 (0.42, 0.61)	0.05	.**000**	0.51 (0.42, 0.61)	0.05	.**000**
Early sleep quality–Late sleep quality	0.43 (0.32, 0.54)	0.05	.**000**	0.42 (0.31, 0.53)	0.05	.**000**
Early no smoking–Late no smoking	0.82 (0.78, 0.86)	0.02	.**000**	0.82 (0.78, 0.86)	0.02	.**000**
Early no alcohol–Late no alcohol	0.44 (0.34, 0.55)	0.05	.**000**	0.44 (0.34, 0.54)	0.05	.**000**
Early well‐being–Late well‐being^c^	Ranging between 0.51–0.58	0.04–0.05	**All .000**	Ranging between 0.49–0.54	0.04–0.05	**All .000**
Cross‐lagged paths						
Early healthy lifestyle score–Late well‐being	0.06 (−0.05, 0.17)	0.06	.312	0.03 (−0.08, 0.15)	0.06	.600
Early physical activity–Late well‐being	0.00 (−0.11, 0.11)	0.06	.987	−0.04 (−0.14, 0.07)	0.05	.508
Early diet–Late well‐being	0.01 (−0.10, 0.12)	0.06	.886	0.01 (−0.10, 0.11)	0.06	.927
Early sleep duration–Late well‐being	0.14 (0.04, 0.25)	0.05	.**009**	0.15 (0.04, 0.25)	0.05	.**005**
Early sleep quality–Late well‐being	0.20 (0.09, 0.31)	0.05	.**000**	0.18 (0.07, 0.28)	0.05	.**001**
Early no smoking–Late well‐being	0.17 (0.07, 0.27)	0.05	.**001**	0.16 (0.06, 0.26)	0.05	.**002**
Early no alcohol–Late well‐being	−0.04 (−0.14, 0.07)	0.05	.503	−0.04 (−0.14, 0.06)	0.05	.461
Early well‐being–Late healthy lifestyle score	0.05 (−0.08, 0.18)	0.07	.476	0.02 (−0.10, 0.15)	0.06	.694
Early well‐being–Late physical activity	0.02 (−0.10, 0.14)	0.06	.721	0.02 (−0.09, 0.14)	0.06	.673
Early well‐being–Late diet	0.01 (−0.10, 0.12)	0.06	.826	−0.00 (−0.11, 0.10)	0.05	.947
Early well‐being–Late sleep duration	0.12 (0.01, 0.23)	0.06	.**029** ^d^	0.11 (−0.00, 0.22)	0.06	.054
Early well‐being–Late sleep quality	0.15 (0.39, 0.27)	0.06	.**009**	0.14 (0.03, 0.25)	0.06	.**014**
Early well‐being–Late no smoking	−0.04 (0.78, 1.70)	0.04	.325	−0.04 (−0.12, 0.03)	0.04	.237
Early well‐being–Late no alcohol	−0.01 (−0.13, 0.10)	0.06	.812	−0.03 (−0.14, 0.09)	0.06	.654

*Note*: Early = in early pregnancy (14–16 weeks gestation), Late = in late pregnancy (28 weeks gestation). Significant p‐values (p < .05) are in bold.

^a^ Only the results of the associations between lifestyle and well‐being are presented here. The healthy lifestyle score, physical activity, diet quality, sleep duration, sleep quality, and well‐being were entered in the models as continuous variables, no smoking and no alcohol as dichotomous categorical variables. The results of the associations with the covariates (maternal BMI and age at recruitment, parity, neighbourhood deprivation, and intervention group) in the adjusted models are not presented to enhance readability.

^b^ All coefficients are standardised.

^c^ Range of values provided for the autoregressive effect between well‐being from early to late pregnancy because this effect was estimated in each path model.

^d^ This *p*‐value became borderline non‐significant after applying Benjamini–Hochberg corrections (corrected *p* = .051).

Comparing the results of the subset of participants with information on change in BMI available from recruitment to late pregnancy (*n* = 152), similar results were observed in terms of direction and significance for most associations. After correction for multiple testing, just one difference was observed between the results of the models that did and did not adjust for change in BMI (in addition to the other covariates). The cross‐lagged path between well‐being in early pregnancy and physical activity in late pregnancy was no longer significant after adjustment for change in BMI (Coeff. = 0.17, corrected *p* = .064). All results are presented in Tables S11 and S12.

## DISCUSSION

This study aimed to explore the associations between maternal lifestyle and psychological well‐being in early and late pregnancy in women with overweight/obesity using cross‐lagged panel analysis to test directionality of relationships cross‐sectionally and over time in pregnancy. Significant autoregressive effects were observed for the HLS, individual components, and well‐being from early to late pregnancy. Well‐being was positively correlated with HLS, physical activity, and sleep variables within time points. Sleep and no smoking in early pregnancy predicted higher well‐being in late pregnancy. These results were robust to adjustment, sensitivity analyses excluding pregnancy complications, and correction for multiple testing.

The results showed that many women in this study did not adhere to recommended lifestyle behaviour in pregnancy: only 33% of participants had a high HLS (score 4–5) in early pregnancy and 36% in late pregnancy. The HLS provides a useful tool to obtain a general impression of women's lifestyle behaviour during pregnancy and demonstrates in this study that there is still room for improvement among the participants. Moreover, the results of the cross‐lagged path model analysis showed significant autoregressive effects for all variables analysed from early to late pregnancy. This may indicate that women engaging in less favourable lifestyle behaviours in early pregnancy may continue to do so in late pregnancy, with possible negative consequences for birth and offspring outcomes (Avşar et al., 2021; Chen et al., 2021). Lifestyle interventions during pregnancy can be useful to positively change women's behaviour, such as diet and physical activity, as was shown in the PEARS study (Kennelly et al., 2018) and other pregnancy intervention studies (McGowan et al., 2013; Poston et al., 2015). Nevertheless, it may be worthwhile to start lifestyle interventions even earlier, before conception, to create healthy habits and a more favourable intra‐uterine environment from the start of pregnancy (Philippe et al., 2022).

Well‐being in early pregnancy was also significantly associated with well‐being in late pregnancy. The results showed that the average well‐being score in early pregnancy was slightly lower (56.3%) than in late pregnancy (60.7%). These scores are similar to well‐being scores observed in other Irish pregnancy intervention studies (Yelverton, Geraghty, et al., 2022). The difference in scores observed between early and late pregnancy aligns with the ‘U‐shaped curve’ for well‐being that has been described in the literature (Newham & Martin, 2013). This curve reflects the lower well‐being of women in the first and last trimester of pregnancy, when they are faced with morning sickness, uncertainty (first trimester), and physical ailments and anxiety for birth (third trimester). In the second trimester, well‐being is higher as there are less physical ailments, more certainty about the viability of the pregnancy, and often no change in occupational role yet (Newham & Martin, 2013). In the PEARS study, well‐being was measured with the WHO‐5 which reflects feelings and activities in the past 2 weeks. This spanned thus approximately weeks 12–14 of gestation which marks the end of the first trimester, and weeks 26–28 of gestation which marks the very start of the third trimester and likely reflects the second trimester better than the third. Unfortunately, no data were available on well‐being at other time points in pregnancy in the PEARS study; this could have been interesting to evaluate trajectories of well‐being throughout pregnancy in a more detailed manner.

Despite these ‘typical’ fluctuations in well‐being throughout pregnancy, our autoregressive results show the importance of monitoring and discussing well‐being from early pregnancy on, as it can possibly be an indicator of well‐being in later pregnancy. Moreover, women showing signs of low psychological well‐being or high distress (e.g., depression, anxiety, stress) should be referred for psychological support and monitored closely throughout pregnancy and the postpartum period. Both psychological treatments (e.g., cognitive behavioural therapy) aiming at reducing distress and positive psychology interventions aiming at increasing different components of women's well‐being can be of interest here, as mental health should not merely be considered as the absence of psychological illness (Bassi et al., 2017).

Our results also showed that the HLS, physical activity, and sleep duration and quality were significantly associated with well‐being cross‐sectionally within time points (in early pregnancy and/or in late pregnancy). Researchers from the ROLO study, a randomised control trial of low glycaemic index diet versus no dietary intervention to prevent the recurrence of foetal macrosomia, also found a significant association between well‐being and physical activity but not with diet quality (Horan et al., 2014). In contrast, when examining well‐being in relation to average daily nutrient intake of separate nutrients and nutrient intake according to national recommendations, the researchers of this study did observe significant associations (Yelverton, Rafferty, et al., 2022). Currently, the evidence on the links between diet and well‐being is inconsistent and more research is warranted (Sparling et al., 2017). Regarding physical activity, the evidence is more consistent. Other studies have also reported positive associations between prenatal physical activity and well‐being or quality of life (Campolong et al., 2018; Da Costa et al., 2003). The authors of these studies suggest that physical activity could act as a coping mechanism for psychological stress (Campolong et al., 2018; Da Costa et al., 2003). This could possibly explain why women who reduce their physical activity levels during pregnancy show a reduction in well‐being or quality of life, while women with consistent low levels do not show this reduction (Campolong et al., 2018; Da Costa et al., 2003). In this study, we did not explore the association between changes in levels of physical activity and changes in well‐being scores. This could be of interest though, and could add to the evidence that supports the importance of remaining physically active throughout pregnancy, both for mother and child (Campolong et al., 2018).

Our results demonstrated that sleep duration and sleep quality in early pregnancy predicted well‐being in late pregnancy. In the other direction (well‐being in early to sleep in late pregnancy), these associations were less robust as shown in the sensitivity analyses. Prenatal sleep may thus be an important indicator of well‐being, which aligns with previous research showing that sleep disturbances in the perinatal period are associated with various negative mental health outcomes, such as mood disturbances and higher risks for perinatal depression and anxiety (Bei et al., 2019). Sleep disturbances are common in pregnancy, with 66%–94% of pregnant women reporting alterations in sleep, largely influenced by the dramatic hormonal, physiologic, and metabolic changes that accompany this period (Pien & Schwab, 2004). Nevertheless, sleep problems in pregnancy are often underdiagnosed and undertreated (Bacaro et al., 2020). Because of its associations with well‐being and other health outcomes, sleep disturbances should be taken seriously, carefully monitored, and treated when needed. Cognitive behavioural therapy for insomnia (CBT‐I) is considered an effective first‐line treatment for insomnia and related problems by US and European authorities (Qaseem et al., 2016; Riemann et al., 2017); however, it has not been tested extensively in pregnant populations. A recent systematic review that evaluated interventions for sleep problems during pregnancy called for the urgent need for high‐quality randomised trials as current evidence is very limited (Bacaro et al., 2020). They also urge to improve psychological and sleep health care during pregnancy as this may benefit the mother and child (Bacaro et al., 2020). Similarly, lifestyle interventions during pregnancy aiming at promoting a better health and well‐being of mother and offspring should consider integrating sleep as a factor to monitor and improve, besides common recommendations about diet, physical activity, smoking, and alcohol consumption. This may be beneficial in various ways, as reciprocal and synergetic effects exist between lifestyle factors. It is, for example, known that sleep and physical activity influence each other through complex, bilateral interactions that involve multiple physiological and psychological pathways (Chennaoui et al., 2015).

No smoking was consistently associated with well‐being but surprisingly solely the path between smoking in early pregnancy and well‐being in late pregnancy. It is not surprising that smoking is associated with lower well‐being; previous research has demonstrated that smokers report more daily stressors and more symptoms of anxiety and depression (Paarlberg et al., 1999). It is unclear however why we found no significant concurrent associations in early and/or late pregnancy.

This study focused on a population of pregnant women with overweight/obesity. In Ireland, nearly 50% of pregnant women now have a pre‐pregnancy BMI ≥25 kg/m^2^ (NMH, 2022), making them regular patients in prenatal care settings. Insight into these women's health behaviours and well‐being and related barriers and facilitators is of the utmost importance for ensuring the optimal care of these women and their offspring. The prenatal period is perceived as a window of opportunity to engage in more healthy behaviours, but the right approach is needed. Women with overweight/obesity are commonly confronted with weight stigma in healthcare settings (Ryan et al., 2023) which may inhibit positive change. Anti‐stigma training for healthcare professionals is a step in the right direction. E‐interventions are also promising for empowering women to engage in more healthy behaviours without being subjected to professional judgement and stigma (Langley‐Evans et al., 2022). The high acceptance among women with overweight/obesity of the PEARS diet and exercise intervention with smartphone app confirms this idea (Greene et al., 2021).

Finally, while cross‐lagged path models are proven to be particularly useful for examining the stability and relationships between variables over time (Kearney, 2017), we do not intend to make any claims about causality as a result of this study. Future studies can further investigate causality between lifestyle and well‐being, both in pregnant and non‐pregnant populations. These studies may also consider conceptualising explanatory models for these associations integrating theoretical frameworks.

### Strengths and limitations

This study presents some strengths and limitations. First, the data were derived from an intervention study. This may have influenced the results of the models, even though we adjusted for the intervention group in the analyses. Data were derived from completing participants which may present a selection bias (Hernán et al., 2004). In our study, this appeared to be true as included participants in this study demonstrated more favourable demographics and healthier lifestyle behaviours compared with those excluded (for details, see Table S2). The population was also almost exclusively of white ethnic origin, which may limit the representativeness of our sample and generalisability of the results. Current results were significant but small in size which may limit their clinical relevance. Replication studies with a larger and more diverse sample, not taking part in an intervention study, are required to confirm our results. Despite the use of validated measures, lifestyle behaviours were self‐reported and may be prone to reporting errors or social desirability (Teh et al., 2023). While adjusted models included potential confounders, as with any multivariable analyses, the possibility of unknown, unmeasured, or residual confounding exists. Strengths include the availability of rich data in both early and late pregnancy which allowed us to analyse cross‐lagged panel models and provided novel insights regarding changes in lifestyle and well‐being throughout pregnancy as well as the directionality of these associations. This study is also one of the first studies to integrate sleep data as part of a composite HLS, and–to our knowledge–the first prenatal HLS including sleep data. Finally, the results were mostly robust to sensitivity analyses excluding pregnancy complications, and correction for multiple testing.

## CONCLUSION

A composite HLS, physical activity, no smoking, and sleep duration and quality were associated with a higher well‐being in early and/or late pregnancy. The results highlight the importance of physical activity and sleep in relation to well‐being during pregnancy. Support for women to increase physical activity and appropriate sleep should be provided to enhance prenatal well‐being.

## AUTHOR CONTRIBUTIONS

**Kaat Philippe:** Conceptualization; methodology; writing – original draft; writing – review and editing; formal analysis. **Alexander P. Douglass:** Data curation; writing – review and editing. **Fionnuala M. McAuliffe:** Writing – review and editing. **Catherine M. Phillips:** Conceptualization; methodology; funding acquisition; writing – review and editing.

## CONFLICT OF INTEREST STATEMENT

The authors declare none.

## ETHICS STATEMENT

The authors assert that all procedures contributing to this work comply with the ethical standards of the relevant national and institutional committees on human experimentation and with the Helsinki Declaration of 1975, as revised in 2008.

## Supporting information


Tables S1–S12.

## Data Availability

The data are not publicly available due to privacy or ethical restrictions.
