# Supplementary material for: Associations between lifestyle and well‐being in early and late pregnancy in women with overweight or obesity: Secondary analyses of the PEARS RCT
Source: Br J Health Psychol. 2025 Jan 16;30(1):e12776. doi: 10.1111/bjhp.12776 (PMC11739547; doi:10.1111/bjhp.12776)
Supplement: Supplementary file 1 — Tables S1–S12. [file BJHP-30-0-s001.pdf]

SUPPORTING INFORMATION

Associations between lifestyle and well-being in early and late pregnancy in women with overweight or obesity: secondary analyses of the PEARS RCT

Table of content:

S1: Flow chart of the participants in the PEARS trial and included in the secondary data analysis. 2

S2: Characteristics of included versus excluded participants in this secondary analysis 3

S3: Participants numbers (N; %) for healthy lifestyle scores in early and late pregnancy 4

S4: Participants (N) with a change in healthy lifestyle score from early to late pregnancy. 4

S5: Participants (N) with changes in meeting recommendations for lifestyle behaviours and well-being from early to late pregnancy. 4

S6: Correlation matrix. 5

S7: Visualisation of the models analysed (covariates not depicted) 6

S8: Equation level goodness of fit: R-squared values for unadjusted and adjusted models, indicating how well the variables in the model predict the well-being and lifestyle in late pregnancy.................. 7

S9: Comparison of the standardised coefficients of the cross-lagged paths in the models..................... 8

S10: Associations between the covariates and the lifestyle and well-being variables in the adjusted cross-lagged path models (main analyses, n=387)... 9

S11. Sensitivity analyses of cross-lagged path models, comparing adjusted models with and without additionally controlling for change in BMI from recruitment to late pregnancy (28 weeks gestation) (n=152).. 11

S12: Associations between change in BMI and the lifestyle and well-being variables in the adjusted cross-lagged path models (sensitivity analyses, n=152) 12

S1: Flow chart of the participants in the PEARS trial and included in the secondary data analysis.

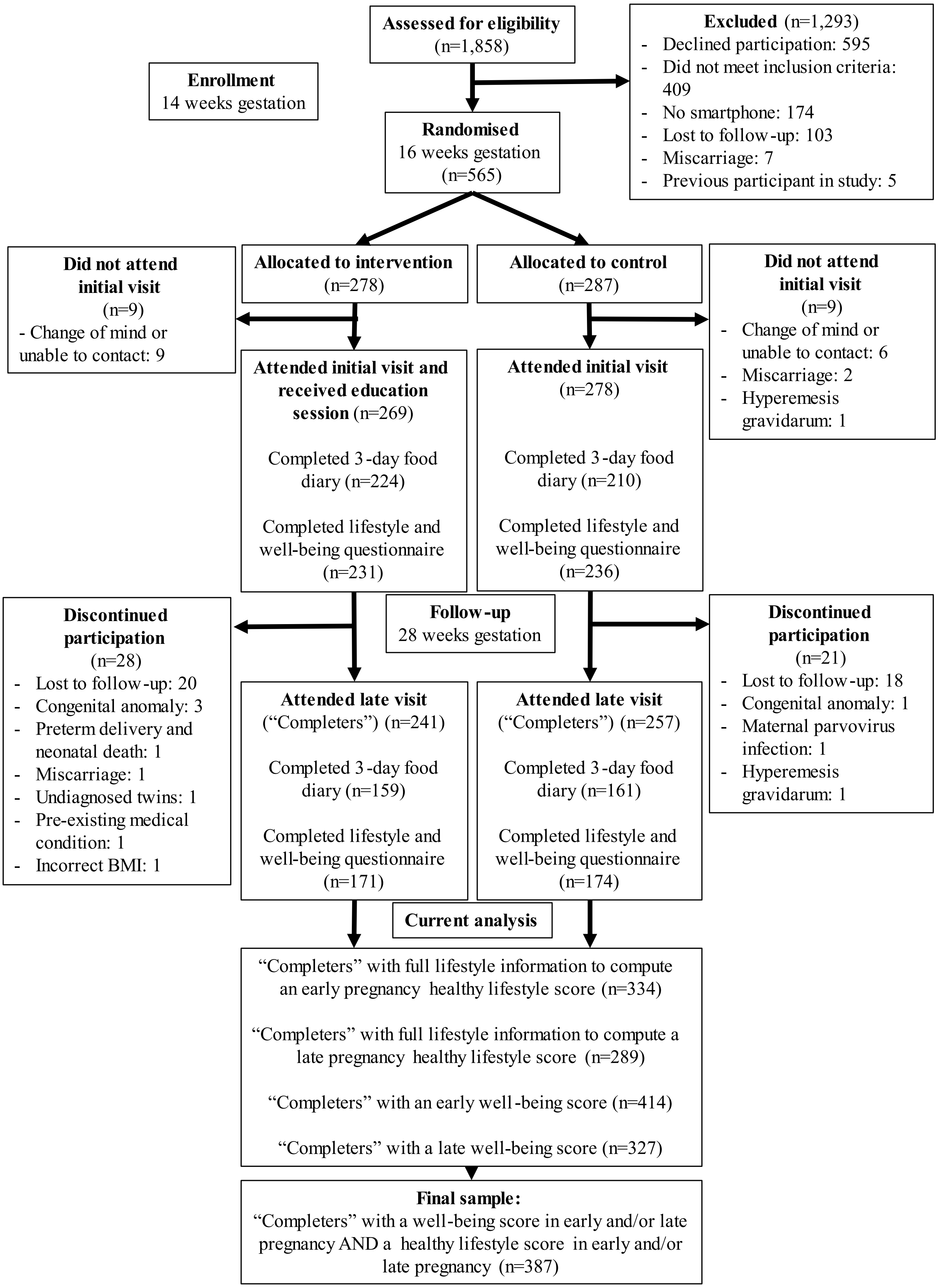

S2: Characteristics of included versus excluded participants in this secondary analysis

	Included participants (n=387)		Excluded participants (n=178)	
	**Total N with data**	**M ± SD or %**	**Total N with data**	**M ± SD or %**
**Maternal characteristics:**				
Age at recruitment	387	32.6 ± 4.2	167	32.2 ± 4.9
Baseline BMI (kg/m^2)	387	29.3 ± 3.3	178	29.1 ± 3.7
Having previous child(ren)	387	45.0%	177	55.4%
Tertiary education or higher	387	66.7%	152	55.9%
Living in advantaged area[†]	387	41.9%	178	39.3%
White ethnicity	375	94.1%	176	90.3%
In intervention group	387	49.6%	178	48.3%
**Lifestyle and well-being scores in early pregnancy:**				
Not currently smoking	368	95.4%	97	91.8%
No alcohol consumption in past 3 months	358	61.7%	90	60.0%
Diet quality: AHEI-P[‡]	361	54.6 ± 10.5	73	51.4 ± 11.2
Physical activity in MET-minutes	368	510.3 ± 444.7	99	508.0 ± 554.9
Sleep duration in minutes	343	436.7 ± 73.0	65	441.4 ± 94.0
Sleep quality[§]	357	2.9 ± 0.7	91	2.8 ± 0.8
Healthy lifestyle score	330	3.1 ± 1.0	31	2.8 ± 1.1
Well-being score[¶]	359	56.7 ± 15.1	89	56.3 ± 17.1
**Lifestyle and well-being scores in late pregnancy:**				
Not currently smoking	312	95.2%	31	93.6%
No alcohol consumption in past 3 months	308	76.3%	31	77.4%
Diet quality: AHEI-P[‡]	305	55.0 ± 10.7	15	51.0 ± 15.2
Physical activity in MET-minutes	313	535.1 ± 396.5	32	528.8 ± 434.3
Sleep duration in minutes	302	392.9 ± 75.3	23	374.35 ± 89.0
Sleep quality[§]	312	2.6 ± 0.7	32	2.4 ± 0.8
Healthy lifestyle score	287	3.1 ± 1.0	5	2.6 ± 0.9
Well-being score[¶]	303	61.0 ± 14.6	29	61.2 ± 16.7

Footnotes: †: Living in an advantaged area is defined as having a HP index above 10; MET = metabolic equivalents of task; ‡: AHEI-P = alternate healthy eating index – adapted for pregnancy with scores ranging from 0-100 with higher scores indicating a higher diet quality; §: sleep quality is a categorical variable (1-2-3-4) with higher scores indicating a better self-perceived sleep quality; ¶: well-being score ranging from 0-100 with higher scores indicating a higher well-being;.

S3: Participants numbers (N; %) for healthy lifestyle scores in early and late pregnancy

	Early pregnancy (n=330)		Late pregnancy (n=287)	
	**N participants:**	**%**	**N participants:**	**%**
**Healthy lifestyle score:**				
**0**	1	0.30	1	0.35
**1**	20	6.06	12	4.18
**2**	65	19.70	74	25.78
**3**	136	41.21	96	33.45
**4**	74	22.42	80	27.87
**5**	34	10.30	24	8.36

S4: Participants (N) with a change in healthy lifestyle score (scored 0-5) from early to late pregnancy.

	**N participants:**
**Change in healthy lifestyle score:**	
**-2**	17
**-1**	49
**0 (no change in score)**	86
**+1**	58
**+2**	16
**+3**	4
**Total N:**	230

S5: Participants (N) with changes in meeting recommendations for lifestyle behaviours and well-being from early to late pregnancy.

	N participants:						
	**Smoking**	**Alcohol consumption**	**Physical activity (500 MET min)**	**Diet quality (top 40%)**	**Sleep (7-9 hours)**	**Sleep quality**	**Well-being**
**Change:**							
**Deterioration**	1	16	34	78	78	91	89
**No change***	289	214	202	161	161	152	20
**Improvement**	3	49	58	19	19	39	167
**Total N:**	293	279	294	258	230	230	275

Footnotes: *"No change" can mean two things: a) participants were not meeting recommendation and this remained unchanged, or b) participants met recommendation and this remained unchanged.; Physical activity and diet were intervention components.

S6: Correlation matrix

	Early well-being	Late well-being	Early HLS	Late HLS	Early no smoking	Late no smoking	Early no alcohol	Late no alcohol	Early PA	Late PA	Early diet	Late diet	Early sleep min.	Late sleep min.	Early sleep quality	Late sleep quality	Group	Previous children	HP-index	BMI	Age
**Early well-being**	1.00																				
**Late well-being**	0.57*	1.00																			
**Early HLS**	0.27*	0.21*	1.00																		
**Late HLS**	0.14*	0.27*	0.42*	1.00																	
**Early no smoking**	0.10	0.26*	0.30*	0.24*	1.00																
**Late no smoking**	0.07	0.24*	0.33*	0.33*	0.86*	1.00															
**Early no alcohol**	0.01	-0.06	0.48*	0.09	0.01	0.04	1.00														
**Late no alcohol**	-0.02	-0.08	0.16*	0.31*	-0.03	0.02	0.46*	1.00													
**Early PA**	0.26*	0.16*	0.45*	0.18*	0.04	0.04	0.01	0.00	1.00												
**Late PA**	0.14*	0.18	0.30*	0.51*	0.03	0.07	0.02	0.02	0.44*	1.00											
**Early diet**	0.09	0.08	0.45*	0.26*	0.06	0.03	-0.02	-0.05	0.04	0.05	1.00										
**Late diet**	0.04	0.09	0.27*	0.46*	0.17*	0.20*	0.00	-0.04	0.07	0.10	0.46*	1.00									
**Early sleep min.**	0.15*	0.22*	0.29*	0.23*	0.12*	0.12*	-0.05	-0.07	0.02	0.11	0.06	-0.00	1.00								
**Late sleep min.**	0.17*	0.35*	0.19*	0.46*	0.12*	0.11*	-0.10	-0.07	0.08	0.22*	0.04	-0.01	0.51*	1.00							
**Early sleep quality**	0.33*	0.36*	0.23*	0.29*	0.05	0.06	-0.05	-0.14*	0.05	0.13*	0.16*	0.19*	0.44*	0.34*	1.00						
**Late sleep quality**	0.24*	0.45*	0.15*	0.32*	0.14*	0.18*	-0.12*	-0.14*	0.03	0.17*	0.08	0.11	0.19*	0.52*	0.42*	1.00					
**Group**	0.06	0.13*	-0.04	0.15*	-0.02	-0.02	-0.03	-0.05	0.00	0.18*	-0.02	0.19*	-0.06	0.10	0.05	0.06	1.00				
**Previous children**	0.08	0.22*	0.20*	0.17*	0.12*	0.13*	0.03	0.14*	0.14*	0.22*	0.06	0.03	0.04	0.08	0.11*	0.16*	-0.05	1.00			
**HP-index**	-0.03	-0.03	0.12*	0.08	0.11*	0.09	-0.02	-0.07	0.04	0.11*	0.08	0.12	-0.10	-0.03	0.02	0.04	0.00	0.17*	1.00		
**BMI**	-0.11*	-0.15*	-0.12*	-0.14*	-0.02	-0.09	-0.01	-0.04	-0.11*	-0.08	-0.07	-0.02	-0.11*	-0.14*	-0.06	-0.20*	0.03	-0.10	-0.06	1.00	
**Age**	0.05	0.03	-0.02	-0.03	0.10	0.08	-0.12*	-0.20*	-0.09	-0.17*	0.17*	0.16*	-0.03	-0.11	0.01	-0.01	0.10*	-0.27*	0.08	0.03	1.00

Notes: HLS = healthy lifestyle score; Early = in early pregnancy (14-16 weeks gestation), Late = in late pregnancy (28 weeks gestation); * $p < 0.05$; group: 1= control group, 2= intervention group; previous children: 1= previous children, 2= no previous children

S7: Visualisation of the models analysed (covariates not depicted)

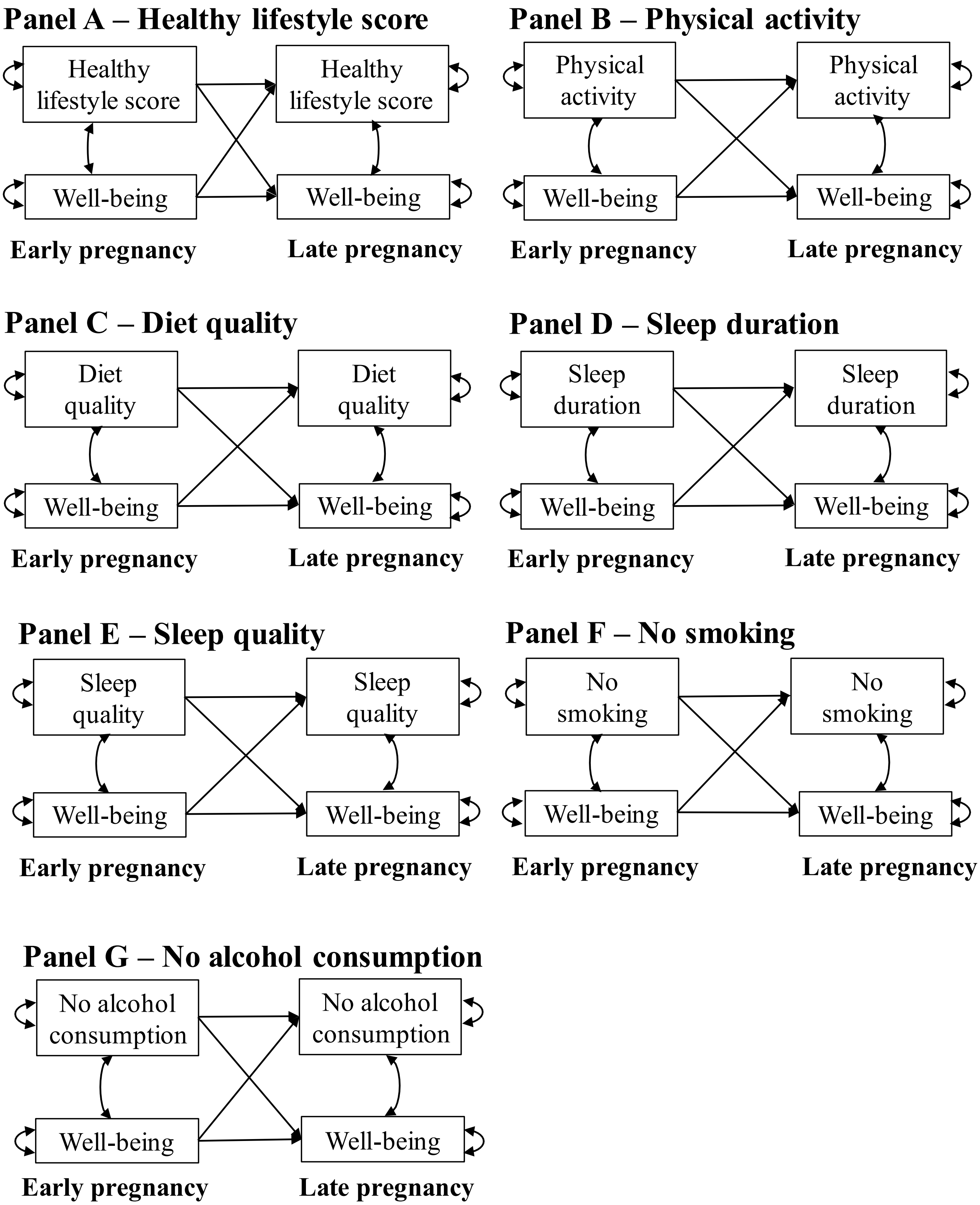

S8: Equation level goodness of fit: R-squared values for unadjusted and adjusted models, indicating how well the variables in the model predict the well-being and lifestyle in late pregnancy:

		Unadjusted path models	Adjusted path models
		R^2	R^2
**Panel A**	Healthy lifestyle score	0.1858	0.2512
	Well-being	0.3435	0.3921
**Panel B**	Physical activity	0.1983	0.2652
	Well-being	0.3368	0.3881
**Panel C**	Diet quality	0.2277	0.2872
	Well-being	0.3406	0.3917
**Panel D**	Sleep duration	0.2928	0.3198
	Well-being	0.3572	0.4068
**Panel E**	Sleep quality	0.2018	0.2431
	Well-being	0.3682	0.4095
**Panel F**	No smoking	0.7135	0.7173
	Well-being	0.3697	0.4144
**Panel G**	No alcohol consumption	0.2253	0.2685
	Well-being	0.3413	0.3922

Note: The healthy lifestyle score, physical activity, diet quality, sleep duration, sleep quality, and well-being were entered in the models as continuous variables, no smoking and no alcohol as dichotomous categorical variables. Adjusted path models were adjusted for maternal BMI and age at recruitment, parity, neighbourhood deprivation, and intervention group.

S9: Comparison of the standardised coefficients of the cross-lagged paths in the models (with estat stdize function):

		Unadjusted path models		Adjusted path models	
**Model**	**Comparison of the cross-lagged paths**	**Chi2**	**p-value**	**Chi2**	**p-value**
**Panel A**	Early healthy lifestyle score → Late well-being vs Early well-being → Late healthy lifestyle score	0.10	0.7501	0.15	0.7011
**Panel B**	Early physical activity → Late well-being vs Early well-being → Late physical activity	0.21	0.6430	0.73	0.3916
**Panel C**	Early diet quality → Late well-being vs Early well-being → Late diet quality	0.01	0.9386	0.22	0.6384
**Panel D**	Early sleep duration → Late well-being vs Early well-being → Late sleep duration	0.04	0.8359	0.15	0.7018
**Panel E**	Early sleep quality → Late well-being vs Early well-being → Late sleep quality	0.67	0.4124	0.55	0.4568
**Panel F**	Early no smoking → Late well-being vs Early well-being → Late no smoking	**13.78**	**0.0002**	**12.61**	**0.0004**
**Panel G**	Early no alcohol consumption → Late well-being vs Early well-being → Late no alcohol consumption	0.09	0.7592	0.05	0.8230

Note: Early = in early pregnancy (14-16 weeks gestation), Late = in late pregnancy (28 weeks gestation); The healthy lifestyle score, physical activity, diet quality, sleep duration, sleep quality, and well-being were entered in the models as continuous variables, no smoking and no alcohol as dichotomous categorical variables. Adjusted path models were adjusted for maternal BMI and age at recruitment, parity, neighbourhood deprivation, and intervention group.

S10: Associations between the covariates and the lifestyle and well-being variables in the adjusted cross-lagged path models (main analyses, n=387)

Association		Adjusted[†] path models		
		**Estimate[‡] (95% CI)**	**SE**	**p-value**
Group →	Early healthy lifestyle score	-0.03 (-0.13, 0.08)	0.06	**0.000**
	Late healthy lifestyle score	0.20 (0.10, 0.30)	0.05	**0.000**
	Early physical activity	0.00 (-0.10, 0.10)	0.05	0.988
	Late physical activity	0.19 (0.09, 0.28)	0.05	**0.000**
	Early diet	-0.02 (-0.12, 0.08)	0.05	0.709
	Late diet	0.19 (0.10, 0.29)	0.05	**0.000**
	Early sleep duration	-0.04 (-0.15, 0.06)	0.05	0.412
	Late sleep duration	0.11 (0.02, 0.21)	0.05	**0.020**
	Early sleep quality	0.06 (-0.04, 0.17)	0.05	0.230
	Late sleep quality	0.01 (-0.09, 0.11)	0.05	0.795
	Early no smoking	-0.00 (-0.10, 0.10)	0.05	0.967
	Late no smoking	0.00 (-0.06, 0.06)	0.03	0.951
	Early no alcohol	-0.03 (-0.13, 0.07)	0.05	0.541
	Late no alcohol	0.01 (-0.08, 0.11)	0.05	0.790
	Early well-being[§]	All 0.08	0.05	Ranging between 0.103 – 0.115
	Late well-being[§]	Ranging between 0.07 – 0.09	0.05	Ranging between 0.053 – 0.126
Previous → children	Early healthy lifestyle score	0.20 (0.09, 0.30)	0.05	**0.000**
	Late healthy lifestyle score	0.08 (-0.03, 0.19)	0.06	0.162
	Early physical activity	0.14 (0.04, 0.24)	0.05	**0.005**
	Late physical activity	0.10 (0.01, 0.21)	0.05	**0.038**
	Early diet	0.32 (0.22, 0.42)	0.05	**0.000**
	Late diet	0.04 (-0.07, 0.14)	0.05	0.477
	Early sleep duration	0.04 (-0.06, 0.14)	0.05	0.452
	Late sleep duration	0.02 (-0.08, 0.12)	0.05	0.701
	Early sleep quality	0.11 (0.01, 0.21)	0.05	**0.037**
	Late sleep quality	0.08 (-0.03, 0.18)	0.05	0.151
	Early no smoking	0.12 (0.02, 0.22)	0.05	**0.016**
	Late no smoking	0.03 (-0.04, 0.09)	0.03	0.400
	Early no alcohol	0.03 (-0.07, 0.13)	0.05	0.581
	Late no alcohol	0.12 (0.02, 0.23)	0.05	**0.018**
	Early well-being[§]	Ranging between 0.07 – 0.08	0.05	Ranging between 0.139 – 0.152
	Late well-being[§]	Ranging between 0.17 – 0.19	0.05	**All 0.000**
HP-index →	Early healthy lifestyle score	0.14 (0.04, 0.24)	0.05	**0.008**
	Late healthy lifestyle score	-0.01 (-0.12, 0.09)	0.05	0.801
	Early physical activity	0.05 (-0.06, 0.15)	0.05	0.372
	Late physical activity	0.10 (-0.00, 0.19)	0.05	0.051
	Early diet	0.32 (0.22, 0.42)	0.05	**0.000**
	Late diet	0.07 (-0.03, 0.16)	0.05	0.188
	Early sleep duration	0.04 (-0.06, 0.14)	0.05	0.461
	Late sleep duration	0.05 (-0.14, 0.05)	0.05	0.360
	Early sleep quality	0.02 (-0.08, 0.12)	0.05	0.674
	Late sleep quality	0.01 (-0.09, 0.11)	0.05	0.860
	Early no smoking	0.13 (0.03, 0.23)	0.05	**0.009**
	Late no smoking	-0.04 (-0.10, 0.02)	0.03	0.178
	Early no alcohol	-0.02 (-0.12, 0.08)	0.05	0.684
	Late no alcohol	-0.11 (-0.21, -0.02)	0.05	**0.019**
	Early well-being[§]	All -0.04	0.05	Ranging between 0.453 – 0.485
	Late well-being[§]	Ranging between -0.05 – -0.07	0.05	Ranging between 0.104 – 0.273

Baseline → BMI	Early healthy lifestyle score	-0.11 (-0.22, -0.01)	0.05	**0.040**
	Late healthy lifestyle score	-0.08 (-0.18, 0.03)	0.05	0.160
	Early physical activity	-0.11 (-0.21, 0.01)	0.05	**0.032**
	Late physical activity	-0.02 (-0.12, 0.07)	0.05	0.637
	Early diet	0.32 (0.22, 0.42)	0.05	**0.000**
	Late diet	-0.01 (-0.11, 0.09)	0.05	0.865
	Early sleep duration	-0.10 (-0.20, 0.01)	0.05	0.068
	Late sleep duration	-0.11 (-0.21, -0.01)	0.05	**0.027**
	Early sleep quality	-0.06 (-0.16, 0.05)	0.05	0.287
	Late sleep quality	-0.18 (-0.28, 0.08)	0.05	**0.000**
	Early no smoking	-0.04 (-0.13, 0.07)	0.05	0.507
	Late no smoking	-0.04 (-0.10, 0.02)	0.03	0.210
	Early no alcohol	-0.02 (-0.12, 0.08)	0.05	0.684
	Late no alcohol	-0.00 (-0.10, 0.10)	0.05	0.978
	Early well-being§	Ranging between -0.10 – -0.11	0.05	**Ranging between 0.035 – 0.042**
	Late well-being§	Ranging between -0.08 – -0.10	0.05	**Ranging between 0.024 – 0.046**
Age →	Early healthy lifestyle score	0.24 (0.13, 0.35)	0.06	**0.000**
	Late healthy lifestyle score	-0.01 (-0.12, 0.10)	0.06	0.897
	Early physical activity	-0.09 (-0.19, 0.01)	0.05	0.092
	Late physical activity	-0.11 (-0.22, -0.01)	0.05	**0.028**
	Early diet	0.32 (0.22, 0.42)	0.05	**0.000**
	Late diet	0.08 (-0.03, 0.18)	0.05	0.144
	Early sleep duration	-0.10 (-0.21, 0.00)	0.05	0.052
	Late sleep duration	-0.06 (-0.16, 0.04)	0.05	0.241
	Early sleep quality	0.01 (-0.09, 0.12)	0.05	0.789
	Late sleep quality	0.00 (-0.10, 0.11)	0.05	0.925
	Early no smoking	0.10 (-0.00, 0.20)	0.05	0.053
	Late no smoking	-0.00 (-0.07, 0.06)	0.03	0.972
	Early no alcohol	-0.11 (-0.21, -0.01)	0.05	**0.028**
	Late no alcohol	-0.08 (-0.18, 0.02)	0.05	0.119
	Early well-being§	Ranging between 0.05 – 0.06	0.05	Ranging between 0.282 – 0.318
	Late well-being§	Ranging between 0.02 – 0.06	0.05	Ranging between 0.234 – 0.750

Footnotes: Early = in early pregnancy (14-16 weeks gestation), Late = in late pregnancy (28 weeks gestation); The healthy lifestyle score, physical activity, diet quality, sleep duration, sleep quality, and well-being were entered in the models as continuous variables, no smoking and no alcohol as dichotomous categorical variables; ‡: All coefficients are standardised. §: Range of values provided for the autoregressive effect between well-being from early to late pregnancy because this effect was estimated in each path model. Group: 1= control group, 2= intervention group; previous children: 1= previous children, 2= no previous children.

S11. Sensitivity analyses of cross-lagged path models, comparing adjusted models with and without additionally controlling for change in BMI from recruitment to late pregnancy (28 weeks gestation) (n=152)

	Adjusted path models			Adjusted path models adjusted for change in BMI		
	**Estimate‡ (95% CI)**	**SE**	**p-value**	**Estimate‡ (95% CI)**	**SE**	**p-value**
**CORRELATIONS WITHIN TIME-POINTS**						
Early healthy lifestyle score – Early well-being	0.37 (0.23, 0.51)	0.07	**0.000**	0.37 (0.23, 0.52)	0.07	**0.000**
Early physical activity – Early well-being	0.29 (0.14, 0.44)	0.08	**0.000**	0.29 (0.14, 0.44)	0.08	**0.000**
Early diet – Early well-being	0.15 (-0.01, 0.31)	0.08	0.065	0.15 (-0.01, 0.31)	0.08	0.064
Early sleep duration – Early well-being	0.14 (-0.03, 0.30)	0.08	0.102	0.14 (-0.02, 0.31)	0.08	0.086
Early sleep quality – Early well-being	0.41 (0.27, 0.54)	0.07	**0.000**	0.41 (0.28, 0.55)	0.07	**0.000**
Early no smoking – Early well-being	0.08 (-0.10, 0.25)	0.09	0.408	0.07 (-0.10, 0.25)	0.09	0.414
Early no alcohol – Early well-being	0.08 (-0.08, 0.25)	0.08	0.306	0.08 (-0.08, 0.24)	0.08	0.323
Late healthy lifestyle score – Late well-being	0.19 (0.01, 0.37)	0.09	**0.043**¶	0.18 (-0.01, 0.36)	0.09	0.061
Late physical activity – Late well-being	0.17 (-0.02, 0.36)	0.10	0.077	0.18 (-0.01, 0.37)	0.10	0.063
Late diet – Late well-being	0.12 (-0.06, 0.31)	0.10	0.198	0.13 (-0.06, 0.32)	0.10	0.178
Late sleep duration – Late well-being	0.25 (0.07, 0.42)	0.09	**0.005**	0.22 (0.04, 0.40)	0.09	**0.015**
Late sleep quality – Late well-being	0.34 (0.18, 0.49)	0.08	**0.000**	0.33 (0.16, 0.49)	0.08	**0.000**
Late no smoking – Late well-being	0.22 (0.04, 0.40)	0.09	**0.016**	0.22 (0.04, 0.40)	0.09	**0.019**
Late no alcohol – Late well-being	-0.21 (-0.38, -0.03)	0.09	**0.021**	-0.21 (-0.39, -0.04)	0.09	**0.018**
**AUTOREGRESSIVE EFFECTS OVER TIME**						
Early healthy lifestyle score – Late healthy lifestyle score	0.40 (0.21, 0.60)	0.10	**0.000**	0.40 (0.21, 0.60)	0.10	**0.000**
Early physical activity – Late physical activity	0.37 (0.22, 0.53)	0.08	**0.000**	0.39 (0.23, 0.54)	0.08	**0.000**
Early diet – Late diet	0.55 (0.42, 0.67)	0.06	**0.000**	0.55 (0.42, 0.67)	0.06	**0.000**
Early sleep duration – Late sleep duration	0.41 (0.26, 0.56)	0.08	**0.000**	0.42 (0.27, 0.56)	0.08	**0.000**
Early sleep quality – Late sleep quality	0.38 (0.21, 0.55)	0.09	**0.000**	0.38 (0.21, 0.55)	0.09	**0.000**
Early no smoking – Late no smoking	0.84 (0.78, 0.90)	0.03	**0.000**	0.84 (0.78, 0.90)	0.03	**0.000**
Early no alcohol – Late no alcohol	0.45 (0.30, 0.60)	0.08	**0.000**	0.45 (0.29, 0.60)	0.08	**0.000**
Early well-being – Late well-being§	Ranging between 0.50 – 0.58	0.06 – 0.07	**All 0.000**	Ranging between 0.51 – 0.59	0.06 – 0.07	**All 0.000**
**CROSS-LAGGED PATHS**						
Early healthy lifestyle score – Late well-being	-0.00 (-0.18, 0.17)	0.09	0.957	0.01 (-0.17, 0.19)	0.09	0.943
Early physical activity – Late well-being	-0.01 (-0.17, 0.15)	0.08	0.883	-0.03 (-0.19, 0.13)	0.08	0.728
Early diet – Late well-being	-0.11 (-0.26, 0.05)	0.08	0.165	-0.12 (-0.27, 0.04)	0.08	0.140
Early sleep duration – Late well-being	0.14 (-0.01, 0.29)	0.08	0.066	0.15 (0.00, 0.30)	0.08	**0.044**¶
Early sleep quality – Late well-being	0.17 (0.00, 0.33)	0.08	**0.048**¶	0.17 (0.00, 0.33)	0.08	**0.049**¶
Early no smoking – Late well-being	0.16 (0.03, 0.29)	0.07	**0.015**	0.15 (0.02, 0.28)	0.07	**0.020**
Early no alcohol – Late well-being	-0.04 (-0.19, 0.12)	0.08	0.640	-0.05 (-0.20, 0.10)	0.08	0.514

Early well-being – Late healthy lifestyle score	0.03 (-0.16, 0.21)	0.10	0.783	0.04 (-0.15, 0.23)	0.10	0.697
Early well-being – Late physical activity	0.18 (0.02, 0.33)	0.08	**0.023**	0.17 (0.01, 0.32)	0.08	**0.032¶**
Early well-being – Late diet	0.06 (-0.08, 0.21)	0.07	0.440	0.06 (-0.09, 0.20)	0.05	0.440
Early well-being – Late sleep duration	0.15 (-0.02, 0.31)	0.08	0.077	0.17 (0.01, 0.33)	0.08	**0.035¶**
Early well-being – Late sleep quality	0.11 (-0.06, 0.28)	0.09	0.193	0.13 (-0.04, 0.29)	0.09	0.134
Early well-being – Late no smoking	-0.03 (-0.13, 0.08)	0.05	0.623	-0.02 (-0.12, 0.08)	0.05	0.697
Early well-being – Late no alcohol	-0.19 (-0.34, -0.04)	0.08	**0.014**	-0.18 (-0.34, -0.03)	0.08	**0.020**

Footnotes: Early = in early pregnancy (14-16 weeks gestation), Late = in late pregnancy (28 weeks gestation). †: Only the results of the associations between lifestyle and well-being are presented here. The healthy lifestyle score, physical activity, diet quality, sleep duration, sleep quality, and well-being were entered in the models as continuous variables, no smoking and no alcohol as dichotomous categorical variables. The results of the associations with the covariates (maternal BMI and age at recruitment, parity, neighbourhood deprivation, intervention group, and change in BMI) in the models are not presented to enhance readability. The additional associations between change in BMI and well-being and lifestyle are presented in table S12. ‡: All coefficients are standardised. §: Range of values provided for the autoregressive effect between well-being from early to late pregnancy because this effect was estimated in each path model. ¶: These p-values became non-significant after applying Benjamini-Hochberg corrections (corrected p's ranging from 0.064 to 0.086).

S12: Associations between change in BMI and the lifestyle and well-being variables in the adjusted cross-lagged path models (sensitivity analyses, n=152)

		Adjusted† path models		
**Association**		**Estimate‡ (95% CI)**	**SE**	**p-value**
Change in BMI →	Late healthy lifestyle score	-0.07 (-0.25, 0.10)	0.09	0.408
	Late physical activity	0.08 (-0.07, 0.24)	0.08	0.287
	Late diet	0.04 (-0.11, 0.19)	0.08	0.572
	Late sleep duration	-0.20 (-0.36, -0.03)	0.08	**0.018**
	Late sleep quality	-0.15 (-0.31, 0.01)	0.08	0.066
	Late no smoking	-0.05 (-0.15, 0.06)	0.05	0.373
	Late no alcohol	-0.06 (-0.22, 0.10)	0.08	0.482
	Late well-being§	Ranging between -0.07 – -0.09	0.07 – 0.08	Ranging between 0.226 – 0.347

Footnotes: Late = in late pregnancy (28 weeks gestation); The healthy lifestyle score, physical activity, diet quality, sleep duration, sleep quality, and well-being were entered in the models as continuous variables, no smoking and no alcohol as dichotomous categorical variables; ‡: All coefficients are standardised. §: Range of values provided for the autoregressive effect between well-being from early to late pregnancy because this effect was estimated in each path model
